# Wintertime investigation of PM_10_ concentrations, sources, and relationship with different meteorological parameters

**DOI:** 10.1038/s41598-023-49714-w

**Published:** 2024-01-02

**Authors:** Bahadar Zeb, Allah Ditta, Khan Alam, Armin Sorooshian, Badshah Ud Din, Rashid Iqbal, Muhammed Habib ur Rahman, Ahsan Raza, Mona S. Alwahibi, Mohamed S. Elshikh

**Affiliations:** 1 Department of Mathematics, Shaheed Benazir Bhutto University Sheringal, Dir (Upper), 18000 Khyber Pakhtunkhwa Pakistan; 2 Department of Environmental Sciences, Shaheed Benazir Bhutto University Sheringal, Dir (U), Khyber Pakhtunkhwa, 18000 Pakistan; 3 School of Biological Sciences, The University of Western Australia, 35 Stirling Highway, Perth, WA 6009 Australia; 4 Department of Physics, University of Peshawar, Khyber Pakhtunkhwa, Pakistan; 5 Department of Chemical and Environmental Engineering, University of Arizona, Tucson, AZ 85721 USA; 6 Department of Hydrology and Atmospheric Sciences, University Arizona, Tucson, AZ 85721 USA; 7 University Boys College, Shaheed Benazir Bhutto University Sheringal, Dir (U), Khyber Pakhtunkhwa, Pakistan; 8 Department of Agronomy, Faculty of Agriculture and Environment, The Islamia University of Bahawalpur, Bahawalpur, Pakistan; 9 Department of Seed Science and Technology, Institute of Plant Breeding and Biotechnology, MNS University of Agriculture Multan, Punjab, Pakistan; 10 Institute of Crop Science and Resource Conservation (INRES), Crop Science, University of Bonn, 53115 Bonn, Germany; 11 Leibniz Centre for Agricultural Landscape Research (ZALF), Eberswalder Straße 84, 15374 Müncheberg, Germany; 12 Department of Botany and Microbiology, College of Science, King Saud University, 11451 Riyadh, Saudi Arabia

**Keywords:** Environmental sciences, Atmospheric science

## Abstract

Meteorological factors play a crucial role in affecting air quality in the urban environment. Peshawar is the capital city of the Khyber Pakhtunkhwa province in Pakistan and is a pollution hotspot. Sources of PM_10_ and the influence of meteorological factors on PM_10_ in this megacity have yet to be studied. The current study aims to investigate PM_10_ mass concentration levels and composition, identify PM_10_ sources, and quantify links between PM_10_ and various meteorological parameters like temperature, relative humidity (RH), wind speed (WS), and rainfall (RF) during the winter months from December 2017 to February 2018. PM_10_ mass concentrations vary from 180 – 1071 µg m^−3^, with a mean value of 586 ± 217 µg m^−3^. The highest concentration is observed in December, followed by January and February. The average values of the mass concentration of carbonaceous species (i.e., total carbon, organic carbon, and elemental carbon) are 102.41, 91.56, and 6.72 μgm^−3^, respectively. Water-soluble ions adhere to the following concentration order: Ca^2+^  > Na^+^  > K^+^  > NH_4_^+^  > Mg^2+^. Twenty-four elements (Al, Si, S, Cl, K, Ca, Ti, V, Cr, Mn, Fe, Co, Ni, Co, Zn, Ga, Ge, As, Se, Kr, Ag, Pb, Cu, and Cd) are detected in the current study by PIXE analysis. Five sources based on Positive Matrix Factorization (PMF) modeling include industrial emissions, soil and re-suspended dust, household combustion, metallurgic industries, and vehicular emission. A positive relationship of PM_10_ with temperature and relative humidity is observed (r = 0.46 and r = 0.56, respectively). A negative correlation of PM_10_ is recorded with WS (r =  − 0.27) and RF (r =  − 0.46). This study’s results motivate routine air quality monitoring owing to the high levels of pollution in this region. For this purpose, the establishment of air monitoring stations is highly suggested for both PM and meteorology. Air quality standards and legislation need to be revised and implemented. Moreover, the development of effective control strategies for air pollution is highly suggested.

## Introduction

A clean environment is a fundamental need for human comfort, health, well-being, and climate^1^. However, both developed and developing countries face air pollution issues with significant impacts, especially on health and climate^2,3^. Particulate matter (PM) is a type of air pollutant and consists of suspended liquid droplets or solid particles in the atmosphere^4^ and is often classified in categories such as smoke, fume, smog, mist, haze, clouds, and fog^5^. Particulate matter comes from anthropogenic activities like fuel burning, oil refineries, automobiles, energy power plants, industrial emissions, and the burning of coal and biomass. Particulate matter also is derived from natural sources like wind-borne dust, sea salt, volcanic emissions, forest fire, wood debris, soil dust, and photochemical and gas-to-particle conversion from biogenic precursor vapors^6^. Particulate matter can be directly released into the atmosphere as particles called primary aerosols, while it can also be generated in the atmosphere by the process of gas-to-particle conversion to generate secondary aerosols^7^. PM_10_ refers to particles with a diameter equal to or less than 10 µm and have been intensely investigated over the past few in recent decades. In many metropolitan areas, it has been claimed that both paved and unpaved roads are essential contributors to the overall mass concentration of (PM_10_). Numerous studies have concluded that traffic-induced re-suspension is the primary cause of coarse particles.

Diverse constituents, for example, organic and inorganic carbon, biological components, inorganic salts (such sodium chloride, ammonium nitrate, and ammonium sulfate), iron compounds, trace metals, and minerals derived from soils, rocks, and building materials generate particulate matter^8^. Carbonaceous species namely organic carbon (OC) and elemental carbon (EC) may have an impact on (1) absorption and scattering efficiencies during interaction with solar radiation, (2) environmental carcinogenicity^9–11^, and (3) bioaerosols^12^. According to Bølling et al.^13^, the key source of EC is the incomplete combustion of fuels and carbon-rich materials, whereas the primary source of OC is biogenic and anthropogenic emissions. Major anthropogenic sources include biomass burning emissions^14^, vehicular emissions^15^, and industrial emissions^16^. Elemental carbon exerts a net heating effect due to its absorptive properties^17^. Moreover, in tropospheric aerosols, water-soluble ions make up the majority of particle matter. The amount and distribution of water-soluble ions can provide information about the sources, atmospheric chemical processes, and potentially harmful outcomes. Water-soluble inorganic species are often substantial contributors to atmospheric particle composition. They are primarily impacted by meteorological variables, geographical circumstances, and particle emissions (e.g., industry, traffic, agriculture, and natural sources)^18^.

Various meteorological parameters like temperature, relative humidity (RH), wind speed (WS), rainfall (RF), as well as atmospheric stability have significant effects on the increase or decrease of PM mass concentration on a local scale^19–21^. The distribution of PM in the atmosphere has an important implication for climate change^22^. That is why it is critical to study meteorological parameters in PM-related studies. In European countries, PM is sensitive to temperature during all seasons^23^, which includes effects on gas-particle partitioning and photochemistry^24^. Wind direction and wind speed (WS) are key parameters, guiding air movement with implications for PM^25,26^. Rainfall (RF) can scavenge and reduce PM^27^. Relative humidity (RH) also has a role in the distribution of air pollution and influences changes in diurnal and seasonal time scales^28,29^. Studies report especially high PM during stable meteorological conditions marked by low boundary layer heights and low wind speed^30^.

In recent years, there has been an emergent concern about almost all levels of aerosols in Pakistan^31^. Likewise, it is of great concern that the concentration and sources of airborne particulates fluctuate prominently with place, season, and meteorological conditions^32^. To save the city from severe pollution, there is a need to make an air quality management policy. Source apportionment of ambient air pollutants helps outline effective air quality management. Receptor modeling ha been used in several studies and proves to be an applicable and proficient tool for source identification of particulate matter in urban or suburban environments^33^. Here, we applied positive matrix factorization (PMF) to identify the various sources of PM in the study region. There is a strong spatiotemporal variation in particulate matter, which requires regular and precise investigation of pollution status, especially in Peshawar city.

The goal of this work is to characterize the mass concentration of ambient PM_10_, carbonaceous species like total carbon (TC), organic carbon (OC), and elemental carbon (EC), and water-soluble ions like NH_4_^+^, Na^+^, K^+^, Ca^2+^, and Mg^2+^ for the urban environment of Peshawar (Pakistan) during the winter season 2018. The study also examines the association between meteorological factors (temperature, relative humidity, rainfall, and wind speed) and ambient PM_10_ concentrations for the study location. Furthermore, an analysis of the sources of PM_10_ in the urban environment by using the PMF model and backward trajectories was conducted for the study area. Our results improve knowledge of the trends of PM_10_ concentrations, carbonaceous species, and water-soluble ions during the winter season. Policymakers will find great use for this data in developing effective air pollution management plans, establishing efficient compliance monitoring, performing epidemiological health research, and putting in place a health warning system in Peshawar (Pakistan).

## Materials and methods

### Description of the study area and meteorological conditions

**Figure 1:**
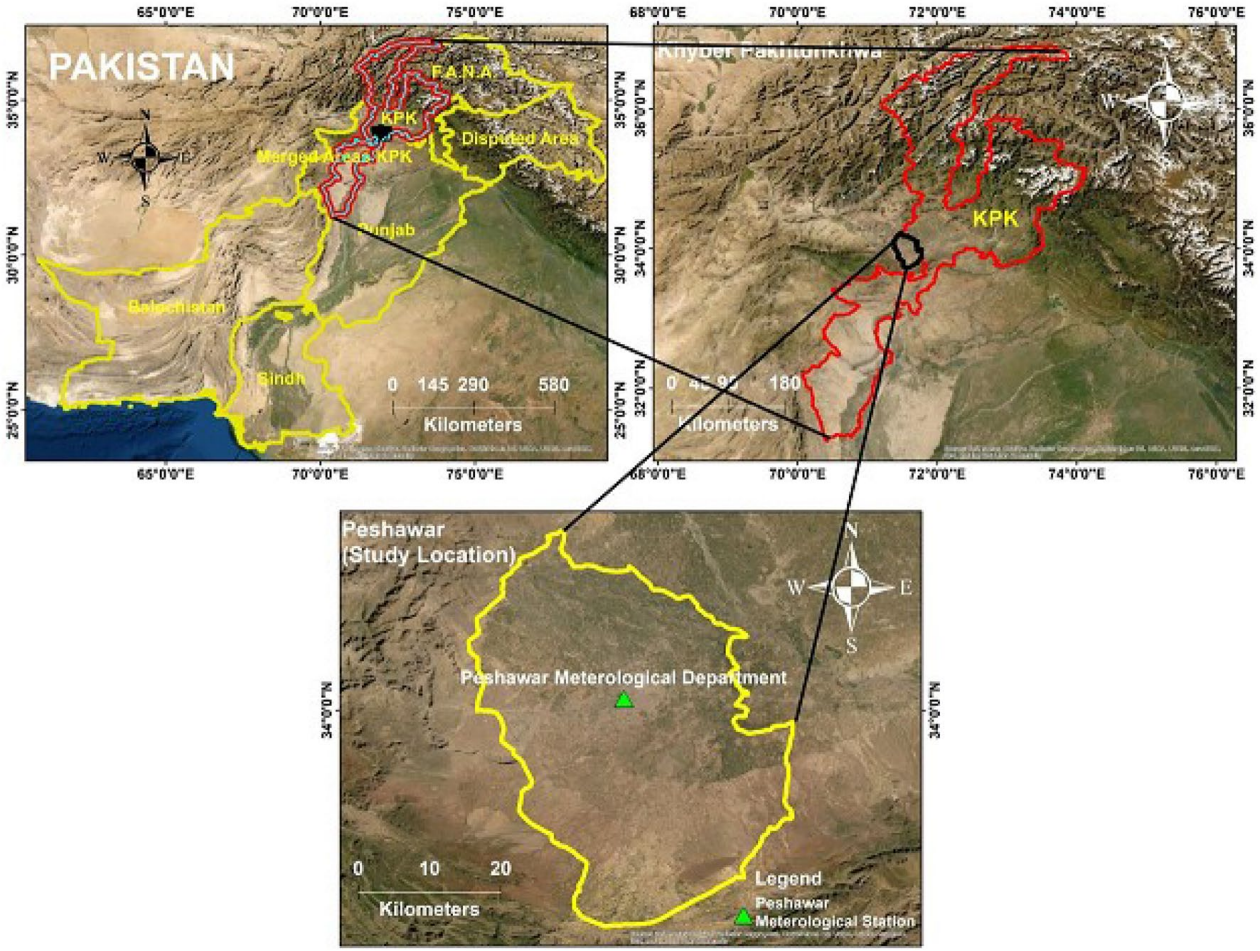
The study's location is shown on a map where samples of particulate matter have been collected.

Peshawar is a megacity located in Pakistan (Fig. 1; 71.56°E, 34.03°N). The city of Peshawar is spread over an area of 1,257 km^2^ having an altitude of 359 m. The population of the city is estimated to be around 4 million people, and growing due to migration to the city in pursuit of employment and various other amenities^34^. Peshawar is an industrial city that produces different types of products like medicine, shoes, cotton, paper, wood goods, steel, cigarettes, iron utensils, flour, and cooking oils^34^. Summer (May–August) and winter (November–March) in Peshawar are hot and cold, respectively. The mean values of maximum and minimum temperatures in this city are 40 °C and 10 °C in the summer and winter, respectively. In Peshawar city, PM_10_ samples were collected at the meteorological center that is operated by the Pakistan Meteorological Department (PMD).

**Figure 2:**
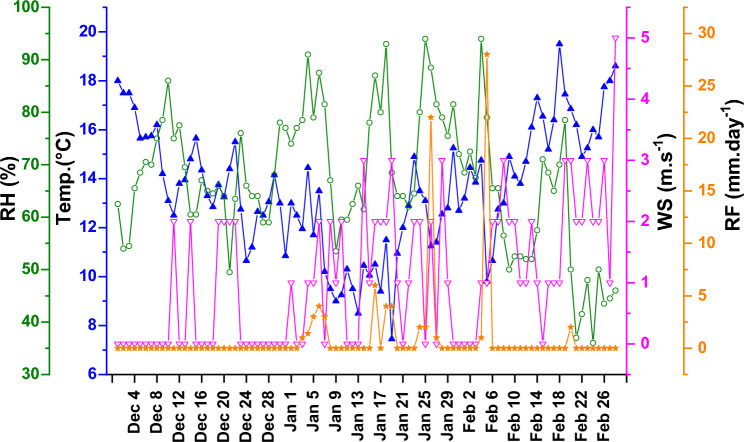
Daily time series of meteorological variables (temperature, relative humidity, wind speed, and rainfall) during the study period.

The Fig. 2 summarizes the daily variation in temperature (°C), RH (%), RF (mm), and WS (m s^−1^). The daily meteorological data used in the present research work were acquired from the PMD center (Peshawar). The average value of temperature is found to be 13.35 ± 2.93 °C with its values varying between 7.05 and 20.75 °C. Wind speed ranges from 0 to 5 m s^−1^ with an average value of 1.04 ± 1.12 m s^−1^. Similarly, the average value of RF is observed to be 1.00 ± 3.83 mm with its values varying from 0 to 28 mm.

### ***Sampling and PM***_***10***_*** measurement***

Particulate matter (PM_10_) samples were collected by the Pakistan Meteorological Department (PMD) in Peshawar. PMD is surrounded by bustling roads, railway lines, bazaars, and small industries. PM_10_ samples were collected for 24 h (8 am to 8 am) on quartz fiber filters. The quartz filter papers used in this study have a diameter of 47 mm along with a pore size of 0.4 µm. The collection of particulate matter samples is carried out with a Low Volume Sampler (LVS) (Tisch Environmental, WILBUR). Ninety samples were collected between December 2017 and February 2018. For the sampling process, the sampler was placed at 5 m height on the rooftop of a PMD building in Peshawar. The flow rate of the sampler was adjusted at 16 L min^−1^. Before any gravimetric measurements, all filters were placed in a balance chamber and allowed to equilibrate for 128 h by keeping constant temperature (21 °C) and relative humidity (33%) regarding winter samples. Filter papers were weighed with a microbalance (Sartorius model MC5, precision: 1 131 µg) to calculate the mass of PM_10_. The individual filter paper was saved inside the aluminum foil and preserved below or at − 20°C in absolute darkness. For the determination of the mass concentration of the OC, EC, organic compounds, and ion species, a portion of the filters was further analyzed. The gravimetric mass (GM) of the collected PM was computed by subtracting the blank filter mass from that of the loaded filter paper mass. The PM concentration was calculated by using the following formula:

$$PM \;mass \;concentration \left( {\mu g m^{ - 3} } \right) = \frac{GM }{{24 \times 60 \times FL }}$$

where GM is the gravimetric mass (µg), FL is the flow rate (L min^−1^), 24 is hours in a day, and 60 is minutes per hour.

### Chemical analysis

The elemental analysis of PM_10_ was done with Particle Induced X-ray Emission (PIXE)^35^. Using the GUPIX software created by Guelph University, the collected spectra of X-rays were examined^36^. Irradiating appropriate micrometer thin target standards were used to calibrate the PIXE system. The PIXE analysis includes the following 24 elements: Al, Si, S, Cl, K, Ca, Ti, V, Cr, Mn, Fe, Co, Ni, Co, Zn, Ga, Ge, As, Se, Kr, Ag, Pb, Cu and Cd. PIXE is an important nuclear analytical technique and has long been used to examine atmospheric aerosols^37–40^. Due to the incident proton beam's precise dimensions, this method is efficient for analyzing small targets in the sample. The PIXE analytical approach is chosen over conventional Energy Dispersive X-Ray Fluorescence because it offers superior sensitivities and lower limits of detection^41^. The PIXE protocol's specifics, the experimental process, and its calibration have all been covered elsewhere^42,43^. In general, a PIXE spectrum will not show elements with an atomic number lower than magnesium (12). Because of this, PIXE is typically better suited for figuring out the heavier metal content than the organic components.

### Operational procedure of PIXE

The experimental setup for PIXE techniques consists of an accelerator, a vacuum chamber with a target holder, an ion beam, and monitoring equipment. For PIXE analysis, the up-to-date Tandem Accelerator Facility (5MV Pelletron, National Electrostatic Corporation, USA) at the National Center for Physics (Islamabad, Pakistan) was used. A stream of powerful ions is used by the Van de Graft accelerator (12 MV) to produce a beam of 2–5 MeV protons that covers a 10 mm-diameter circle. When this beam of protons collides with PM atoms, inner electron shell vacancies are produced. The experimental system was examined regularly for accuracy and repeatability. To adjust the results, the blank filters were also regularly examined. Samples were deposited in the vacuum chamber at 10^−7^ torr and exposed to a 2.5 MeV proton beam with a 2.0 mm^2^ collimator. The samples were positioned at a 90-degree angle concerning the incident beam. The holders allow for the analysis of five samples at a time. To reduce the intensity of low-energy X-rays coming from the matrix elements, a 40 mm thick Mylar absorber foil was positioned in front of the detector. At 5.9 keV, the detector's energy resolution was 129 eV. The samples are placed around 1 cm away from a 500 nm Si_3_N_4_ beam extraction window. A collimator at the end of the beam line sets the beam spot to 1*2 mm^2^ and a graphite Faraday cup placed directly behind the samples measure the charge flow during the experiment.

To account for the variations in the X-ray emission cross sections, the detection system at the time of this analysis relied on two Silicon Drift Detectors (SDD), which were optimized for the detection of low-Z and medium–high-Z elements^44^ The detector used for low-Z elements was a 10 mm^2^ and 280 µm thick Ketek GmbH SDD with a 1 ms shaping time and a 145 eV FWHM energy resolution at the 5.9 keV Mn Ka line. It was collimated to 7 mm^2^ by a Ta-Cr-Ti–Al multilayer collimator to protect the outer region where incomplete charge collection may occur^44,45^ The detector was positioned at a 45-degree angle to the beam line, was protected from backscattered protons by a magnetic deflector, and the volume between its entrance window and the sample was saturated with helium. A 450 µm thick, 113 mm^2^ (collimated to 80 mm^2^) Ketek GmbH SDD with 165 eV FWHM energy resolution at the 5.9 keV Mn Ka line and a 1 ms shaping time was used to identify the medium–high Z elements. This SDD detector was placed at an angle of 135° concerning the direction of the beam. It has a 25 mm thick Be entrance window, and to reduce the low energy X-rays, absorbers (450 mm Mylar foils) were placed in front of it. Using a 10nA current for 300 s, samples were bombarded with a 3.0 MeV proton beam on the target (equivalent to 3.2 MeV in vacuum), which is the ideal beam energy for the examination of quartz fiber filters^46,47^. The majority of the samples’ area was analyzed using a scanning system to average across any potential non-homogeneous deposits. Elements concentrations were determined by comparing the sample yields with a sensitivity curve that was established by measuring a set of thin Micromatter standards (with a 5% uncertainty) under identical experimental conditions.

### Quality assurance procedures

For the quality assurance and quality control (QA/QC) program, it is important to remember that the beam density profile is uniform and better than 0.5% and the proton beam energy is stable to be better than 1 part per 1000. This guarantees that the target sample will produce X-rays consistently and uniformly. A quality control standard, not to exceed once per hour, can be run as an additional assurance of machine stability. Replica analyses can also be carried out and a blank filter has been subtracted from that of the loaded one. EAI also keeps a large library of standards from NIST, USGS, NIES, NBS, and acquisitions that can be utilized for QA/QC. All of these standards' values are either certified or well-established, having already undergone analysis using other accepted methodologies.

### System calibration

For the PIXE machine that bases its calibration on thin film gravimetric standards, the mass/area can be represented as a straightforward ratio of yields. For thin unidentified target:

$$\left( {M/A} \right) \, f \, = \, \left( {Qs/Q} \right)\left( {Y/Ys} \right)\left( {M/A} \right) \, s \, Fs$$

where "y" is the number of X-ray counts in a peak due to the specific element as measured with a given detector, chamber geometry, and absorber; "M/A" denotes the total mass per unit area; "f" is the mass fractional content of the specific element to be analyzed; "Q" is the integrated charge in the proton bombardment; and "s" denotes the corresponding quantity for the standard. A target is considered thin if its thickness exhibits insignificant fluctuations in both X-ray production and self-absorption. Using thin films of evaporated metals or compounds as Micromatter Corporation gravimetric standards of seventy-two elements, an X-ray count against atomic number calibration curve is constructed, normalized per unit mass and proton charge. Periodically, a thorough calibration is carried out to verify that the geometric layout of the bombardment facility is constant and to assess how the aging X-ray detector is changing. A Gallium Phosphide (GaP) standard is run as part of the quality control system at least once a day to account for small fluctuations in calibration drift. Higher atomic number elements produce fewer X-rays per unit proton charge than lower atomic number elements do because X-ray production cross-sections drop with rising atomic number for a given electronic shell and detector efficiency reduces with increasing X-ray energy. Consequently, a twofold irradiation is applied to each sample to counteract this effect and produce more consistent detection limits throughout the whole periodic table. The detector can view the target's X-rays directly in one spot. As a result, the low atomic number of elements can grow. To balance the spectrum and selectively filter the X-rays that are released, an absorber is positioned between the sample and the detector in the second position. Control over the detection limits of individual elements in the spectrum is possible by modification of these irradiation periods. These identical circumstances are used for standards calibration, which ensures that every element has a spectrum in every position. It is possible to extrapolate to any desired combination of irradiation timings for unknown targets using a normalized linear combination of these two places for the standards. To do the calibration, each standard is irradiated in front of the detector—both with and without the filter—for a predetermined charge collection. The relative intensities for each of the X-ray lines are then determined and kept in a library once the standards are fitted into the gravimetric mass. A least squares polynomial fit of the standards is used to establish calibration curves, which are measured in counts/(µgram/cm^2^/µCoulomb). For a given proton energy, the chance of producing X-rays is a smooth continuous function of the atomic number, which justifies this technique. In this way, the gravimetric analysis's minor inaccuracies are evened out and its clear flaws become more noticeable. After these curves are determined for the K and L line X-ray groups, they are saved and integrated with the X-ray line and intensity library to form the system's calibration.

### Validation of the measurement

The GUPIX program was used to analyze the PIXE spectra to determine the absolute concentration^48^. The software was utilized in batch mode to efficiently examine every spectrum. To ensure the absolute concentration, a correction factor was introduced and thin single-element standards put on polycarbonate filters (Micromatter) were examined. The reported concentrations were verified by looking at the NIST SRM 2783 air particles on the reference material of the filter medium. Using SRMs is a well-established technique in analytical chemistry to ensure the accuracy and reliability of analytical findings. While the concentrations of the heavier elements (S through Pb) were determined using proton beam measurements, the light elements (Na, Mg, Al, and Si) were determined using helium beam measurements. The recovery was computed by dividing the analytical results by the certified value.

### Uncertainty

The uncertainty in the amount of the element measured was reduced by using thin samples and improving the uniformity of the particle beam. Both the target sample's thickness and the particle beam's homogeneity are somewhat controllable. However, for the majority of the cases of interest, the elemental distribution in the matrix is generally unknown. This lack of information yields an intrinsic uncertainty in the quantitative elemental estimation, limiting the PIXE method's precision.

### Limits of detection

Through GUPIX analysis, the limits of detection (LOD) for a particular element were determined. For a specific element, this computation is based on three times the square root of the backdrop over one full-width half maximum (FWHM), with the centroid of the primary peak serving as the center of gravity. A higher limit of detection for light elements is made possible by the decrease in the X-ray background in helium studies^49,50^. The limits of detection show that protons are a superior option for heavier elements like K, Ca, Cr, Fe, and Pb, while helium beams are better suited for Al and Si as well.

#### Positive matrix factorization

The positive matrix factorization (PMF) model is a multivariate receptor-based model^51^. The sources as well as their contribution to PM_10_ at the study location are found through this model. PMF model has been widely used for the identification and apportionment of sources of PM^52–55^.

PMF is a non-data-sensitive technique that requires no univariate analysis to resolve inhomogeneous datasets. To address challenging data sets, such as those with outliers or levels below detection limits, PMF may incorporate error estimates, or weights, corresponding to the data. Analytical methods appropriate for the specific medium and significant species needed to distinguish impacts define the composition. A data matrix X of i by j dimensions (i.e., j = chemical species measured with particular uncertainties s_ij_; i = number of samples) can be created from a speciated data set. This receptor model's objective is to solve the chemical mass balance (CMB) between measured species concentrations and source profiles (Eq. (1), taking into account the number of factors (p), each source's species profile (f), and the mass g that each factor contributes to each sample.

1 $$X_{ij} = \mathop \sum \nolimits_{K = 1}^{P} g_{ik}\, f_{kj} + e_{ij}$$

PMF uses the least squares method to analyze source profiles (f_kj_) and contributions (g_ik_). The EPA PMF's goal is to minimize the sum of squares of standardized residuals (Q), which are calculated by dividing the residual by the relevant uncertainty value.

2 $$Q = \mathop \sum \nolimits_{i = 1}^{a} \mathop \sum \nolimits_{j = 1}^{b} \left( {\frac{{e_{ij} }}{{S_{ij} }}} \right)^{2}$$

In the above equation “a” represents the entire quantity of samples collected, “b” represents the overall quantity of species, s_ij_ represents the uncertainty for j-th species in the i-th sample.

and e_ij_ shows the contribution of j-th factor in i-th samples.

where, a = total number of samples, b = total number of species, and s_ij_ = uncertainty for j-th species in the i-th sample.

Equation (1) is defined as

3 $${\text{X }} = {\text{ GF }} + {\text{ E}}$$

where X is the matrix of measured data with dimension “no. of samples” and M “no. of species”. G is the Contributions Matrix with dimension “no. of samples” and M “no. of factors”. F is the source profiles matrix with dimension “no. of species” and M “no. of factors”. E is the matrix of residuals with dimension “no. of samples” and M “no. of species”.

The PMF model takes as inputs the uncertainty matrix "S" and the matrix of the observed concentrations "M," and outputs are matrices "G," "F," and "E.". The measured PM_10_ mass was taken into consideration while applying the source contribution matrix "G" for source apportionment. The model Eq. (1) presents a scaling coefficient, y_k_, for quantitative source apportionment in the following manner

4 $$x_{ij} = \mathop \sum \nolimits_{k = 1}^{p} g_{{ik \frac{{y_{k} }}{{y_{k} }}}} f_{kj} + e_{ij}$$

Therefore using multi-linear regression of the estimated source contribution against the measured particle matter mass, y_k_ was determined. It was considered that zero would be the linear regression constant.

In addition

5 $$m_{i} = \mathop \sum \nolimits_{k = 1}^{m} y_{k } + e_{ij}$$

This method was incorporated into the EPA-PMF version 5.0 and used to categorize the sources and contributions of the PM_10_ samples that were collected at the receptor site based on the analytical data. Following many runs, the five most appropriate sources with the lowest Q value were identified by EPA-PMF 5.0.

#### Pearson correlation analysis

The strength of the connection between two factors can be calculated through correlation analysis, which is usually combined with regression analysis. The Pearson correlation is used to quantify a correlation between at least two continuous variables and is denoted by r. To investigate the correlation of PM_10_ with various meteorological parameters like temperature, RH, WS, and RF, multiple regression analysis was applied. The impacts of several climatic conditions on PM concentration are found during this analysis. The coefficient of each variable was known, so we can estimate the best-fit model as

$$PM = a_{0} + a_{1} Temp. + a_{2} RH + a_{3} WS + a_{4} RF$$

where a_0_ is the intercept and a_1_, a_2_, a_3_, and a_4_ are the regression coefficients of Temp, RH, WS, and RF, respectively.

#### Chemical analysis of carbonaceous species and water-soluble ions

A part of the sample, 1.5 cm^2^, was cut and subjected to thermal/optical analysis on a Carbon Aerosol Analyzer (Sunset Laboratory, Forest Grove). The in-depth process regarding OC-EC analysis is explained by Öztürk and Keleş^56^. The instrument was standardized using a sucrose solution (approximately 3.5 μg μL^−1^). To ensure quality control, the analyzer was calibrated daily with a reference sucrose solution and an empty punch of a pre-heated quartz filter. Concerning blank corrections, the sampled filters were passed through the same analysis too.

The entire filter paper blank concentration for OC and EC was found to be 0.5 ± 0.2 μg cm^−2^ and 0.0 ± 0.02 μg cm^−2^, respectively. The blank concentration of OC and EC were then deduced from that of the mass concentration in the loaded filter paper. The mass concentration of carbonate carbon (CC) was found by manually integrating thermograms between 210–220 s and 270–285 s. Cachier et al.^57^ describe in detail the decarbonizing of the PM sample using HCl vapors and afterward passing an aliquot of the acidified sample. The peaks regarding CC that emerged through the initial stage of the process of heating in the inert atmosphere (100% He) were additionally found.

Confirmation of calibration constancy contains CO_2_ standards, checks (weekly) through sucrose, autocalibration daily, leak checks, system blanks, and checks of laser performance. Every measurement is within ± 10% of the TC. Instruments that go beyond QC limits (± 5%) are promptly pulled down for investigation. If it is discovered that the calibration has changed by more than 5%, all intervening samples are re-analyzed. Lower measurable limits are defined by the variability of dynamic field blanks, while minimum detection limits are set by laboratory blanks. Replicate analyses are used to determine the analytical precisions for each batch of measurements. On different instruments, duplicate analyses (10% of all samples) are carried out. For the determination of cations, a specific part of the filter paper is extracted using 1% nitric acid (v/v). Using a Dionex ICS 1100 Ion Chromatograph, ion chromatography is used to assess the major cations (NH^4+^, Na^+^, K^+^, Ca^2+^, and Mg^2+^). For the analysis of cations, the system contains essential things like (1) a guard column (CG12A, 450 mm), (2) an analytical column (CS12A, 4 250 mm), and (3) a cation self-regenerating suppressor. The utilized eluent is 20.0 mM, methanesulphonic acid. To introduce the samples, the solutions were supplied into the chromatograph with a loop (25-µL). The flow rate of 1.0 mL min^−1^ was kept for both eluents. Calibration is performed using a set of standards that contain either the necessary cations. Using the aforementioned technique, a blank quartz filter paper was extracted, its blank adjustments examined, and its results were subtracted from the concentration of observed cations.

### Quality control and limit of detection of cations

Before the PM_10_ samples were taken, the sampling filters were appropriately conditioned to eliminate any artifacts. Whatman filter sheets were dried for 24 h in desiccators with silica coarse gel before and after sampling, with only the filter's pre- and post-sampling weights recorded. In the laboratory, analytical grade chemicals were used to generate standards for the measurement of cations.

Seven replicate analyses of the standard solution at very low concentrations were used to compute the method detection limits (MDL) for the cations. The MDL was performed as half of three estimates of the SD of the concentrations. The MDL of the cations like Na^+^, NH_4_^+^, K^+^, Ca^2+^, and Mg^2+^ were selected as 0.01, 0.004, 0.018, 0.074, and 0.041 mg/L, respectively. Additionally, blank filters for all the parameters were analyzed using the same method as the sampled filters and added to the measurements. It was found that the lowest ratio of the measured cation of the blank filters was 0.1. Spiking with known concentrations was done to ascertain the analytical instruments' detection efficiency. The samples were transported in premium self-shielding plastic bags to prevent sample contamination from handling.

## Results and discussions

### ***Mass concentration of PM***_***10***_

**Figure 3:**
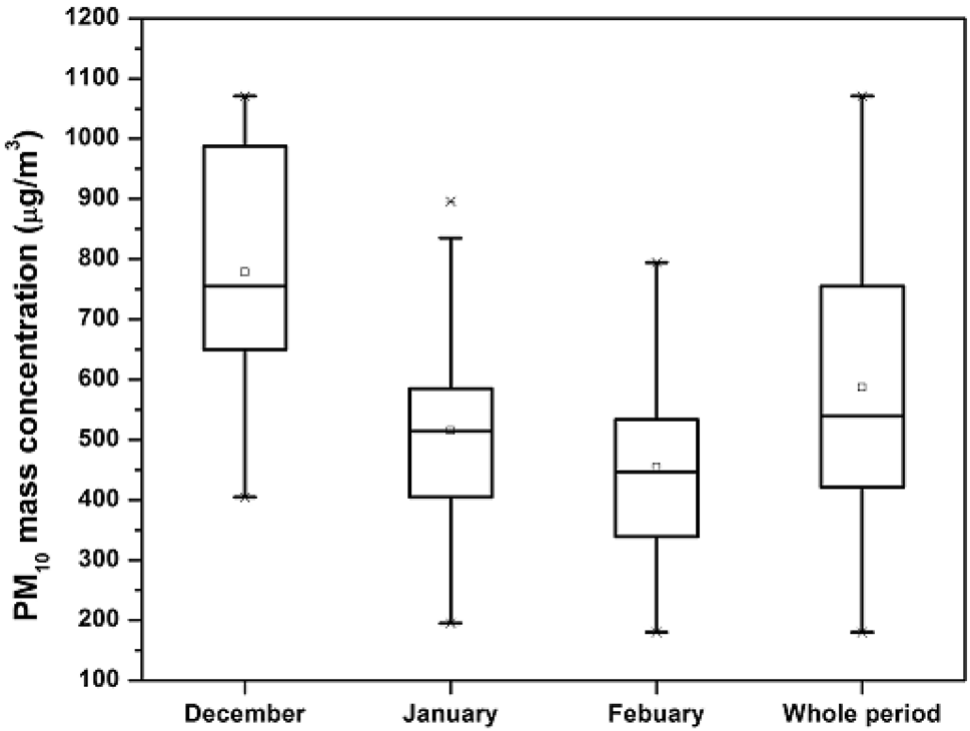
Variation in PM_10_ mass concentration over the study period.

The Fig. 3, presents the average monthly and seasonal fluctuation in the PM_10_ mass concentration in Peshawar, Pakistan. The value of PM_10_ mass concentration during December, January, and February varies from 405 – 1071, 195 – 896, and 180 – 794 µg m^−3^ with an average value of 778 ± 188, 515 ± 166 and 454 ± 139 µg m^−3^, respectively. For the whole study period, the mean value of the mass concentration of PM_10_ is found to be 586 ± 217 µg m^−3^ with its value ranging from the minimum value of 180 to the maximum value of 1071 µg m^−3^. The 24-h mean value of PM_10_ mass concentration was found to be much higher than the standard limit imposed by the World Health Organization (WHO) (45 µg m^−3^)^58^ and National Environmental Quality Standards for Ambient Air (NEQSAA) (150 µg m^−3^)^59^.

The PM_10_ mass concentration is relatively high in December followed by January and February. The high value of the mass concentration of PM_10_ in December is partly due to the absence of precipitation (RF), along with high relative humidity and relatively high-temperature values in addition to calm winds (Fig. 2), which contribute to the high PM_10_ concentrations. There are various causes of increasing PM_10_ mass concentration in winter. The study locations are surrounded by various roads, flyover bridges, railway tracks, residential areas, industries, and bazaars, and thus road dust re-suspension and a variety of anthropogenic emissions sources are suspected to be influential. In Peshawar, the winter season is cold and coincident with low-temperature inversions, and thus PM_10_ accumulates and is trapped near the ground surface.

High PM_10_ concentration is also because of long and short-range transport of aerosols from various locations during the study period (Figure 7). Trajectory results point out that long-range transport of aerosols starts from neighboring countries (e.g., Afghanistan, China, and Kyrgyzstan) during December, January, and February, and reaches the receptor site. The model results also indicate short-range trajectories that originate from local areas with strong influence from anthropogenic emission sources.

The values of PM_10_ mass concentration were also higher than the values measured in other cities of Pakistan. The PM_10_ concentrations over different locations are described in Table 1.

**Table 1:** PM_10_ mass concentration over different locations.

PM 10 concentration	Location	Reference
406 µg m^−3^	Lahore (Pakistan)	34
340 µg m^−3^	Lahore (Pakistan)	60
638 µg m^−3^	Peshawar (Pakistan)	61
438 µg m^−3^	Karachi (Pakistan)	55
34.4 µg m^−3^	Zaragoza (Spain)	62
50.5 µg m^-3^	Ulsan (Korea)	63
39.1 µg m^−3^	Istanbul (Turkey)	64
238.5 µg m^−3^ (urban)	Nowshera (Pakistan)	65
505.1μg m^−3^ (industrial)		
255.0 μg m^−3^ (suburban)		
64 µg m^−3^	Mingoara (Pakistan)	66
284 µg m^−3^	Lahore (Pakistan)	67
279	Lahore (Pakistan)	68
68.2to280.6 µg m^−3^ (residential)	Kolkata (India)	69
62.4 to 401 µg m^−3^ (industrial)	Kolkata (India)	69
238 ± 106 μg m^−3^	Delhi (India)	70
241 µg m^−3^ (winter)	Delhi (India)	71
131.3 µg m^−3^	Dhaka (Bangladesh)	72
136 µg m^−3^	Urban sites ((Bangladesh)	73
124.57 µg m^−3^	Dhaka (Bangladesh)	74
80 to 397 µg m^−3^	Guangzhou (China)	75
140 µg m^−3^	Beijing-China	76
100 µg m^−3^	Shanghai-China	76
60 µg m^−3^	Taipei-China	76
79.6 μg/m^3^ (cold season)	Tehran (Iran)	77
67.9 μg/m^3^ (warm season)	Tehran (Iran)	77
189μg/m^3^(winter)(normal days)	Ahvaz (Iran)	78
742μg/m^3^(winter)(dusty days)	Ahvaz (Iran)	78
586 µg m^−3^	Peshawar (Pakistan)	Present study

#### Carbonaceous specious and water-soluble ions

**Figure 4:**
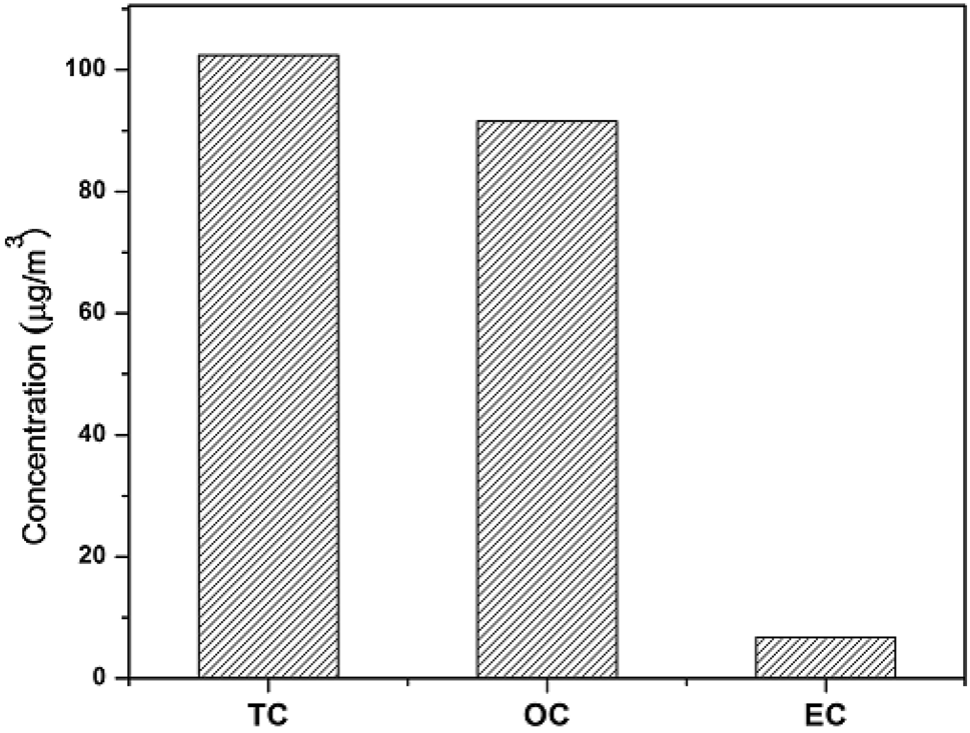
TC, OC, and EC mass concentration values over the study location.

The carbonaceous species (TC, OC, and EC) mass concentrations are shown in Fig. 4. The TC, OC, and EC mass concentration values ranged from 28.98 to 262.24, 26.48 to 251.91, and 1.46 to 11.62 μgm^−3^ with an average value of 102.41, 91.56 and 6.72 μgm^−3^, respectively.

During the study period, the large EC mass concentration value is because of diverse emission sources (e.g., vehicular emission, biomass burning, and coal burning)^60,61^.

Combustion of biomass and fossil fuels, along with secondary organic aerosol formation promote enhanced OC levels in the atmosphere^62^. For context, Zeb et al.^63^ reported the average value of OC and EC over industrial (55.85 and 4.62 μgm^−3^), urban (36.35 and 5.17 μgm^−3^), and suburban (40.05 and 6.33 μgm^−3^) locations of Nowshehra city, Pakistan. Alam et al.^34^ reported OC and EC mass concentration values of 63.42 and 21.15 μgm^−3^, respectively, in Lahore city of Pakistan. Sahu et al.^64^ noted annual mean total carbon (TC) concentration at the urban (46.8 ± 23.8 μg m^−3^) and industrial (98.0 ± 17.2 μg m^−3^) locations over Chhattisgarh (Central India). Satsangi et al.^62^ observed the mass concentration values OC and EC for the four seasons in India: winter (37.4, 6.3 μg m^−3^), post-monsoon (33.0, 3.4 μg m^−3^), summer (29.4, 2.6 μg m^−3^), and monsoon (9.8, 1.7 μg m^−3^).

During the entire period, water-soluble ions followed the following concentration ranking (Table 2): Ca^2+^  > Na^+^  > K^+^  > NH_4_^+^  > Mg^2+^. Ca^2+^ was found to be the main ion with an average mass concentration value of 35.79 ± 17.83 (µg m^−3^), which could be attributed to construction activities going on in the surrounding area of the sampling location. NH_4_^+^ is produced mostly through reactions of NH_3_ with HNO_3_, H_2_SO_4_, and their precursors; NH_3_ is additionally a weak base that reacts with water to yield NH_4_^+^. Generally, the sources of NH_3_ are anthropogenic, particularly the burning of fossil fuels and agricultural activities^65,66^. There is a reduction in agricultural activity during the winter season. However, the production of NH_4_^+^ is more influenced by traffic and coal combustion. Agricultural/animal husbandry, fertilizer, wastewater treatment, ammonium bisulfate, or nitrate, are the sources of the cations, namely ammonium (NH_4_^+^)^67^. Marine/sea salt, dry lakes, and de-icing materials all produce sodium (Na^+^). Magnesium (Mg^2+^) is produced in dry lakes and marine/sea salt. Generally speaking, potassium (K^+^) and calcium (Ca^2+^) are believed to be the markers of biomass combustion and dust, respectively^68^.

**Table 2:** Concentration statistics (minimum, maximum, average, standard deviation) of water-soluble ions.

	Na^+^ (µg m^−3^)	NH_4_^+^ (µg m^−3^)	Mg^2+^ (µg m^−3^)	K^+^ (µg m^−3^)	Ca^2+^ (µg m^−3^)
Min	2.40	0.41	0.68	0.81	9.61
Max	10.65	14.10	4.89	10.70	86.28
Mean	4.98	2.90	2.39	3.71	35.79
Standard deviation	1.53	3.59	0.99	2.17	17.83

Mu et al.^69^ analyzed the PM_10_ concentration for water-soluble ions (i.e., Na^+^, NH_4_^+^, K^+^, Mg^2+^, and Ca^2+^) in Jinzhong (China) and reported their values for winter seasons to be 0.84, 9.00, 0.64, 0.2 and 4.00 µg m^–3^, respectively. Liu et al.^70^ investigated the concentration of Na^+^, NH^4+^, K^+^, Mg^2+^, and Ca^2+^ in PM_10_ at Huangshi (China) having values 5.25, 7.77, 2.10, 0.58, and 5.29, µg m^–3^, respectively. Švédová et al.^71^ noted the mass concentration of EC, NH_4_^+^ Na^+^, K^+^, Ca^2+^, and Mg^2+^ to be 1.26, 3.07, 0.29, 0.34, 0.49, and 0.06 µg m^−3^, respectively, over the Czech Republic. Bhuyan et al.^72^ measured the concentration of Na^+^, NH_4_^+^, K^+^, Ca^2+^, and Mg^2+^ to be 1.3, 1.91, 1.5, 0.60, and 0.1 µg m^–3^, respectively, in PM_10_ concentration over the Brahmaputra valley (India) during the winter season. Kumar et al.^73^ noted the concentration of OC, EC, Na^+^, NH_4_^+^, K^+^, Ca^2+^, and Mg^2+^ in PM_10_ to be 31.5, 15.6, 0.19, 1.31 0.26, 1.12, 0.12 µg m^–3^ at Amritsar (India) and 44, 19.33, 0.28,2.36, 0.53,2.54, and 0.25 µg m^–3^ at Delhi (India). Norazman et al.^74^ analyzed PM_10_ Dhaka (Bangladesh) for water-soluble ions like Na^+^, NH_4_^+^, Mg^2+^, Ca^2+^, and K^+^ and reported values to be 0.14,0.53, 0.07, 1.80 and 0.21 µg m^–3^, respectively. Esmaeilirad et al.^75^ investigated PM_10_ for the OC and EC concentration and reported their values to be 7.8 and 3.5 µg m^–3^ during the winter season in Tehran (Iran). Hassan et al.^76^ investigated that throughout the lockdown period during COVID-19, in Suzhou (China) the percentage concentrations of NH_4_^+^, Ca^2+^, K^+^, and Na^+^ decreased by 48.8, 52, 57 and 76.3%, respectively, in comparison to the pre-COVID ion levels, while Mg^2+^ exhibited an increase of 30.2%. Wang et al^77^ reported that during the Suzhou lock down, the PM_10_, and water-soluble ions decreased by 38.3 and 58.6%, respectively, compared to the pre-COVID period. Jain et al.^78^ investigated the OC and EC concentration in PM_10_ samples having values of 22.7 ± 7.4 and 8.7 ± 3.9 µg m^–3^, respectively, over Indo‑Gangetic Plain.

#### Source apportionment using positive matrix factorization

**Figure 5:**
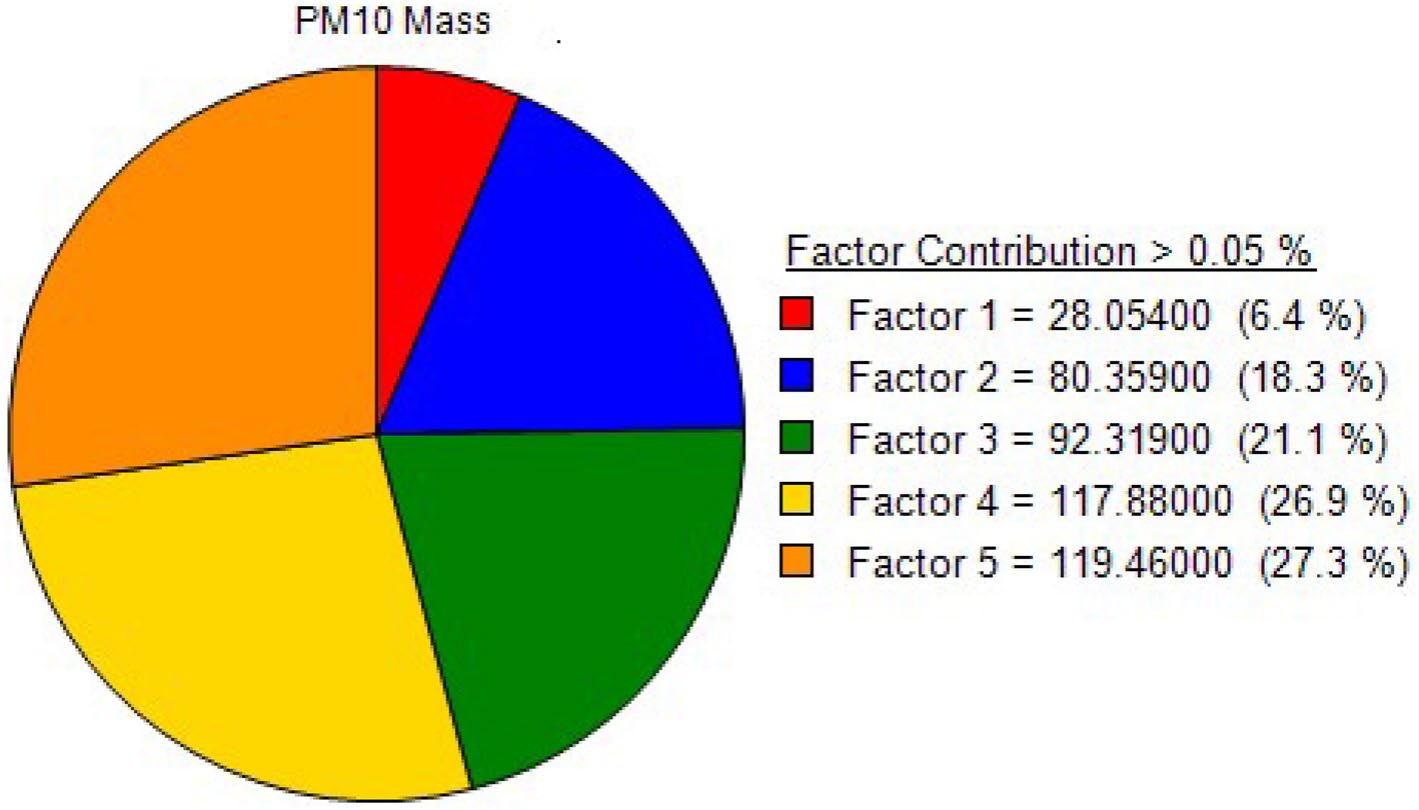
Percentage contributions of recognized sources to PM_10_ in Peshawar, Pakistan.

PM_10_ sources in Peshawar's urban environment were determined using the Positive Matrix Factorization (MF) model version 5.0.14. Different factors were investigated and five optimal numbers were identified (Fig. 5): industrial emission, soil and re-suspended dust, household combustion, metallurgic industries, and vehicular emission. The total contribution of industrial emissions, soil and re-suspended dust, household combustion, metallurgic industries, and vehicular emissions to PM_10_ were 6.4, 18.3, 21.1, 26.9, and 27.3%, respectively. The minimum, maximum, and average elemental concentrations of each element are presented in Table 3.

**Table 3:** Minimum, maximum, and average elemental concentrations of each element.

Elements	Minimum (ppm)	Maximum (ppm)	Mean ± SD (ppm)
Al	17,219	87,079.4	53,381.6 ± 17,159.9
Si	1368.4	27,695.8	18,820.8 ± 7545.1
S	82.6	1867.4	838.7 ± 482.9
Cl	51	4611.3	2097.2 ± 1196.7
K	77.8	9429.6	2801.0 ± 2053.8
Ca	41	48,088	17,684.2 ± 11,293.5
Ti	90.2	1378.5	389.2 ± 284.6
V	2.5	45.6	23.3 ± 12.8
Cr	98.4	2730.3	577.1 ± 643.0
Mn	76.1	586.2	222.9 ± 145.1
Fe	858	16,296.2	4387.6 ± 3346.2
Co	14.9	161.5	52.1 ± 39.1
Ni	8255.6	56,183	30,237.9 ± 10,440.4
Cu	210.9	1464.1	623.2 ± 278.2
Zn	311.8	4003.4	1443.5 ± 944.3
Ga	13.2	197.3	67.7 ± 82.0
Ge	5.4	255.5	97.0 ± 59.3
As	18.7	38	28.3 ± 13.6
Se	6.9	55.8	26.3 ± 13.7
Kr	7.7	101.1	35.2 ± 21.5
Ag	37.8	64.6	50.5 ± 10.3
Cd	45.1	281.5	106.2 ± 90.9
Sn	40.2	61.3	50.1 ± 90.9
Pb	80	400	240 ± 90.9
Cu	70	351	210 ± 90.9

Shahid et al.^56^ studied PM_10_ in Karachi (Pakistan) using the PMF model and identified five possible sources: soil dust, industrial dust, biomass burning, coal combustion, and automobile emissions. Alam et al.^34^ used the PMF model to identify five sources of PM_10_ in Lahore (Pakistan) including brick kiln emissions, residential combustion emissions, re-suspended dust, vehicular emissions, and industrial emissions. According to Gu et al.^79^, there were six PM_10_ sources (resuspended dust, sodium chloride, secondary sulfates, biomass burning, traffic emissions, and secondary nitrate) in Augsburg (Germany). Chan et al.^80^ used the PMF model to identify the primary sources of aerosols in four Australian cities, including combustion, crustal/soil dust, ammonium sulfates, nitrates, motor vehicles, marine aerosols, chloride-depleted marine aerosols, and industry. Similarly, Gupta et al.^81^ utilized the PMF model to identify the sources of PM in Mumbai, India, including traffic, paved road dust, residual oil combustion, and coal-fired boilers and nitrates.

Liu et al.^82^ collected PM_10_ samples from six sites in Tianjin, China, and then carried out a PMF model and identified five sources of PM_10_ to be secondary inorganic aerosols, biomass burning, crustal dust, coal combustion, and vehicle exhaust, which contributed 28–30%, 20–21%, 18– 21%, 17–20%, and 4%, respectively. Koçak et al.^83^ analyzed PM_10_ for water-soluble ions, water-soluble organic carbon, organic and elemental carbon (OC, EC), and trace metals in Istanbul (Turkey) and carried out their source apportionment analysis using Positive Matrix Factorization (PMF). They identified seven factors including secondary, refuse incineration, traffic, fuel oil, solid fuel, crustal, and sea salt. Jain et al.^84^ applied the PMF model for the analysis of PM_10_ to estimate their well-known sources on a seasonal basis in Delhi. They identified eight major sources of PM_10_: secondary nitrate, secondary sulfate, vehicular emissions, biomass burning, soil dust, fossil fuel combustion, sodium and magnesium salts, and industrial emissions. Gupta et al.^85^ investigated seven sources of PM_10_ in Delhi by applying the PMF model. Esmaeilirad et al.^86^ applied the PMF model to PM_10_ in Tehran (Iran) and identified five sources, namely traffic exhaust, biomass burning, industries, sulfate-rich, and nitrate-rich having contributions of 44.5, 6.7, 2, 24.2 and 18.4%, respectively. Begum et al.^87^ used the PMF model and identified eight sources of PM_10_ over Dhaka (Bangladesh) including sea salt, fugitive Pb, two strokes, soil dust, road dust, biomass burning/brick kilns, motor vehicles, and metal smelters. The following are the sources of PM_10_ in Peshawar city identified via the PMF model.

#### Source 1 (Industrial emission)

**Figure 6:**
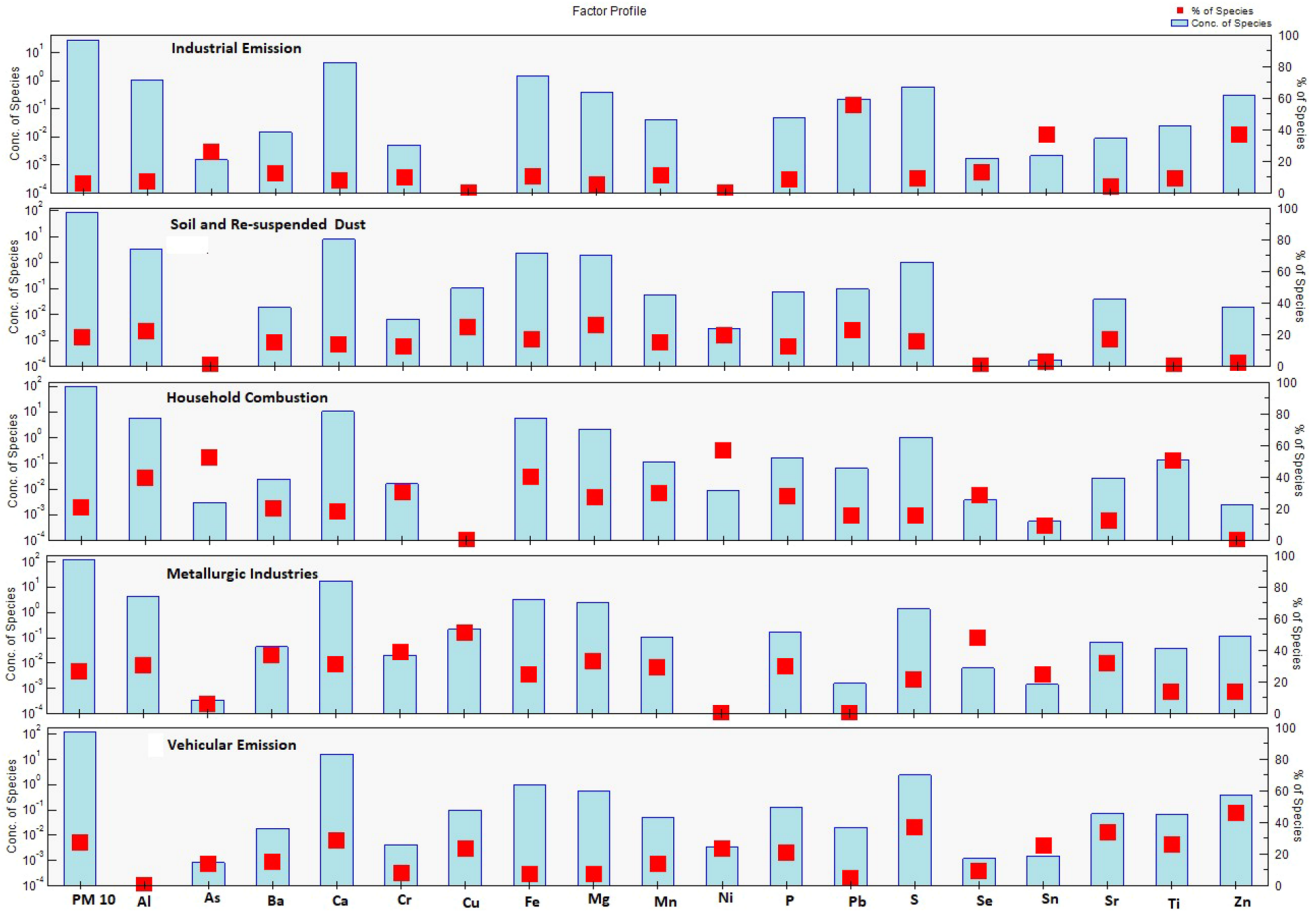
Particulate matter (PM_10_) source composition profiles based on PMF analyses.

The first factor was dominated by elements like Pb, Sn, Zn, and As with minor amounts of Cr, Mn, Fe, P, and Ti (Fig. 6). This factor was thus assigned to industrial emissions. This factor contributed 6.4% of the total mass concentration of PM_10_ as shown in Fig. 5. Lead is one of the major constituents of industrial emissions^88^. In this profile factor, a significant contribution was identified from As, which corresponds to high-temperature processes such as condensation and coagulation of smelting vapors and oil burning^55^. The wide use of combustion heating fuel produces Ni^89^. For metal smelting, various enterprises in Peshawar require gas, dung cakes, rubber, and coal^53,90^. Peshawar also has factories that produce white and brown sugar. Wood, coal, rubber, plastics, and gasoline all produce substantial amounts of smoke, which limits vision and affects air quality^91^. Alam et al.^34^ reported a 12.9% contribution of industrial emissions to the total PM_10_ mass concentration in Lahore.

#### Source 2 (Soil and re-suspended dust)

This source includes the elements Al, Mg, Ca, and K (Fig. 6), which are common components of soil dust^45^. In this factor, the other elements like Cu, Pb, Fe, Zn, and Cr indicate re-suspended road dust during the flow of vehicular and wind. This source contributed about 18.3% to the total PM_10_ mass concentration. Re-suspended road dust was considered the major source of PM in Peshawar city. According to Lough et al.^92^, the elements (Al, Ca, Mg, and Fe) are the primary contributors to coarse particulate matter and Ca is produced from the construction of roads, houses, and buildings. Tire wear increases the concentration of Zn load in road dust and Fe is one of the key components of vehicles^93^. In Peshawar city, most of the land around roads is unpaved and grass-free. In addition, roads are of poor quality, and roadside construction activities result in high loads of re-suspended roadside dust. The low RF during the study period also caused a large contribution of re-suspended dust^94^. Alam et al.^34^ found a dust contribution of 18.2% to PM_10_ mass concentration in Lahore.

#### Source 3 (Household combustion)

The third source identified as household combustion had a significant contribution in As, Ni, Ti, Fe, Pb, S, and Al (Fig. 6). The mean contribution of household combustion was found to be 21.1% to the total mass concentration of PM_10_ (Fig. 5). Household combustion emissions in Peshawar have increased in recent years due to a lack of natural gas, particularly during the winter season, and these emissions primarily contain particles produced from the burning process during cooking and heating. As, S, and Pb concentrations rise as coal and biomass are burned more frequently in Peshawar city. Elements like Ca, Fe, Pb, S, Sr, Ti, Zn, Ni, and minor amounts of Al and Cu are abundant in house combustion^95^. Coal combustion raised the concentrations of Pb, Sr, and Zn in the atmosphere^96^. There are a large number of brick kilns in and around Peshawar that burn coal, rubber, and wood, which causes more As, Zn, and Pb emissions^90^.

#### Source 4 (Metallurgic industry)

This group is characterized by elements like Cu, Se, Mg, P, Sr, Mn, Cr, Ba, and Al and it contributed 26.9% to the total PM_10_ mass concentration (Figs. 5 and 6). The sampling location is surrounded by a large number of metal enterprises, and the main industrial zone is about 5 km to the west of the sampling site. Iron, steel, aluminum, pharmaceuticals, food, rubber, and paint are just a few of the industries in the study area. Cr is one of the significant indicators of emissions from the iron and steel industry. Mansha et al.^55^ reported that the steel and iron industry contributes a high amount of PM_10_. Querol et al.^97^ found the maximum concentration of Cu in PM_10_ in the urban environment of Spain at an industrial site (ceramic and petrochemical industries). Fe and metallurgical emissions produce Zn whose concentration is higher than other materials^88^. Among the main industrial sources of Zn are electroplating, ore processing, smelting, and drainage from both active and inactive mining activities. The elements Cr and Cu are produced in iron smelting factories^98^. Elements like Cu, Zn, and Ni, which are often utilized in the steel and metal mechanics sectors, were found in all samples. CO_2_ is the predominant component of this source, however, certain smelting emissions also consist of the elements like Al, Ca, Cr, Zn, Fe, Mg, Pb, Sr, Ti, Zn, and Ni^90^.

#### Source 5 (Vehicular emissions)

The last source was dominated by S, Zn, Ca, Cu, Sr, and Ti, and corresponded to vehicular emissions with a contribution of 27.3% to the total mass concentration of PM_10_ (Figs. 5 and 6). This source contribution is considered the highest as compared to other sources because the sampling site is surrounded by transportation corridors including busy roads and railways. In Peshawar city, people rely heavily on motorbikes and motorcycles continuously, which are significant sources of Zn because of lubricating oil combustion in the associated engines^90^. In Peshawar city due to narrow roads and various check posts, the traffic is usually slow and congested resulting in high vehicular emissions. The burning of diesel in vehicle engines produces significant amounts of lead (Pb) and sulfur (S) in the environment, which are particularly detrimental to human health. Vehicle emissions contribute significantly to air pollution in urban areas^55,99,100^. According to Alam et al.^34^, automotive emissions generated 27.4% of the total PM_10_ mass concentration in Peshawar. Balakrishna et al.^101^ reported a mean vehicular concentration of 24.92% in Shinjung (Taiwan). Klimaszewska et al.^102^ investigated that in Peshawar city a high amount of Zn is produced by substandard tire quality (i.e. tire wear) and fuel combustion in the engines of various vehicles.

#### Correlation between PM_10_ and meteorological parameters

Climate factors like temperature, relative humidity, wind speed, and rainfall have a direct impact on particulate matter mass concentrations. These meteorological parameters can affect the transport, dispersion, removal (dry and wet deposition), diffusion, and dilution of PM. Meteorological variables also influence air chemistry and, as a result, secondary PM generation^103,104^. The association between PM_10_ concentration OC, EC, and climatic data throughout the study period was determined using Pearson correlations, and the results are displayed in Table 4.

**Table 4:** Pearson correlation between meteorological variables and PM_10_, OC, and EC.

	PM_10_	Temp	RH	WS	RF
PM_10_	1	0.75	0.63	− 0.27	− 0.46
OC	0.67	− 0.77	0.51	0.32	− 0.41
EC	0.52	− 0.48	0.23	0.58	0.37

Where Temp = Temperature (°C), RH = Relative humidity, WS = Wind speed and RF = Rainfall.

PM_10_ concentration exhibited a positive correlation (r = 0.75) with temperature during the study period as shown in Table 4. This positive correlation between atmospheric temperature and coarse PM during the winter season could be explained by the climatic characteristics of the season and the interactions of these parameters with PM. High temperature dries up the earth's surface, lifting loose material from the earth's surface (with sufficient wind and disruption) and consequently can increase PM concentration. Similarly, high temperatures may cause more favorable conditions for atmospheric dispersion as compared to low air temperatures. In addition, most of the land in Peshawar is bare and arid; therefore, high temperatures may cause wind turbulence and re-suspension of dust particles. El-Sharkawy & Zaki^105^ reported positive correlations of PM with temperature in the eastern province of Saudi Arabia. Tai et al.^22^ reported a positive relationship between PM_10_ and temperature in the United States of America. In Makah, Saudi Arabia, Munir et al.^106^ noted a positive link between PM_10_ and temperature. Sirithian et al.^107^ investigated the positive correlation of PM_10_ with temperature (r = 0.528) in Thailand. Gupta et al.^108^ reported a negative correlation of PM_10_ with temperature (r =  − 0.73) in Bangladesh. Pateraki et al.^109^ observed that days with higher temperatures showed larger increases in the concentrations of PM_10_ in an urban Mediterranean area of India.

The mass concentration of PM_10_ in association with relative humidity is depicted in Table 4. The relationship between RH and PM_10_ mass concentrations was found to be positive (r = 0.63). Greater humidity in the atmosphere can enhance aqueous processing to generate larger particles and lead to larger particles via hygroscopic growth^110,111^. Al-Taai & Al-Ghabban^112^ also found a positive correlation between RH and PM_10_ concentration in Baghdad city. Sirithian et al.^107^ investigated the negative correlation of PM_10_ with RH (r =  − 0.600) in Thailand. Gupta et al.^108^ reported a negative correlation of PM_10_ with RH having a correlation coefficient of − 0.73 in Bangladesh. According to Pateraki et al.^109^, there is a negative correlation between humidity and PM increment; that is, as humidity rises, PM_10_ and PM_2.5_ decrease. Munir et al.^106^ identified a negative correlation of RH with PM_10_ (r =  − 0.30) in Makah, Saudi Arabia*.* Kliengchuay et al.^113^ noted a negative correlation between RH and PM_10_ in Thailand from 2009 to 2019.

Wind speed also plays an important role in affecting the mass concentration of PM_10_. During the study period, PM_10_ and WS were shown to be negatively correlated, with a correlation coefficient of − 0.27. The increase in WS results in an increase in the horizontal dispersion of pollutants and, consequently, PM mass concentration drops owing to dilution^114^. Peshawar has a flat topography and hence experiences horizontally homogenous wind flow, which does not allow accumulation of PM. The fact that wind disperses and carries the PM away is the cause of the negative association between WS and PM_10_. In the city of Patras, Karagiannidis et al.^115^ discovered a negative connection between PM_10_ concentration and WS. Li et al.^9^ reported a negative correlation coefficient (− 0.35) between PM_10_ and WS in the metropolitan area of the Sichuan Basin. Kliengchuay et al.^116^ noted a negative association (r =  − 0.14) between PM_10_ and WS in Lamphun (Thailand). Sirithian et al.^107^ investigated a very weak negative correlation of PM_10_ with WS (r =  − 0.037) in Thailand. Sin et al.^117^ noted that the air is always made drier by wind, and the amount of PM drops. Ravindra et al.^118^ claimed that a major factor in the reduction of PM was the movement of air masses.

Rainfall is an important factor in maintaining atmospheric composition. PM_10_ and RF were shown to be negatively correlated (r =  − 0.46) (Table 4). The mass concentration of atmospheric PM is reduced by washout induced by rain. On the other hand, lower RF causes PM to stay in the atmosphere for a longer time, hence increasing its concentration. According to Wang and Ogawa^119^, RF could effectively remove atmospheric PM. RF helps in the removal of coarse PM in ambient air through wet deposition and washout processes^9^. Huang et al.^120^ also found similar types of results in Beijing, China where RF was negatively correlated with PM. Kayes et al.^121^ also reported a negative correlation of RF with PM in the urban environment of Dhaka, Bangladesh. Gupta et al.^108^ reported a negative correlation of PM_10_ with rainfall having a correlation coefficient of (− 0.61) in Bangladesh. Farooq et al.^122^ reported a moderate negative correlation of PM_10_, with WS (− 0.34), a strong negative correlation with temperature (− 0.69) and rainfall (− 0.63), and a weak relationship with RH (− 0.32) in the urban environment of Mingora (Pakistan). In Nigeria, Owoeda et al.^123^ found a negative connection between PM_10_ and RF. A similar trend of PM_10_ and RF was found in Morogoro (Tanzania) where low PM_10_ mass concentrations were found during precipitation events^76^. Li et al.^36^ noted a negative correlation (r =  − 0.59) between PM_10_ and RF in an urban area of the Sichuan Basin. It is found that PM_10_ exhibits a positive correlation with both OC and EC, having a correlation coefficient of 0.67 and 0.52, respectively. OC and EC have a negative correlation with temperature having a correlation coefficient of − 0.77 and − 0.48, respectively. OC and EC have a positive correlation with RH having a correlation coefficient of 0.51 and 0.23, respectively. OC and EC have a positive correlation with WS having correlation coefficients of 0.58 and 0.32, respectively. Similarly, OC and EC have a negative correlation with RF having correlation coefficients of − 0.41 and − 0.37, respectively. Sonwani et al.^124^ reported negative correlations of OC and EC with temperature (r =  − 086, and 0.41) and positive correlations of OC and EC with RH (r = 0.48, 0.14) in Delhi, India. Peng et al.^125^ identified a positive correlation of OC and EC with wind speed having correlation coefficients of 0.62 and 0.04, respectively, at Chongqing City, Southwest China.

#### Trajectory analysis

**Figure 7:**
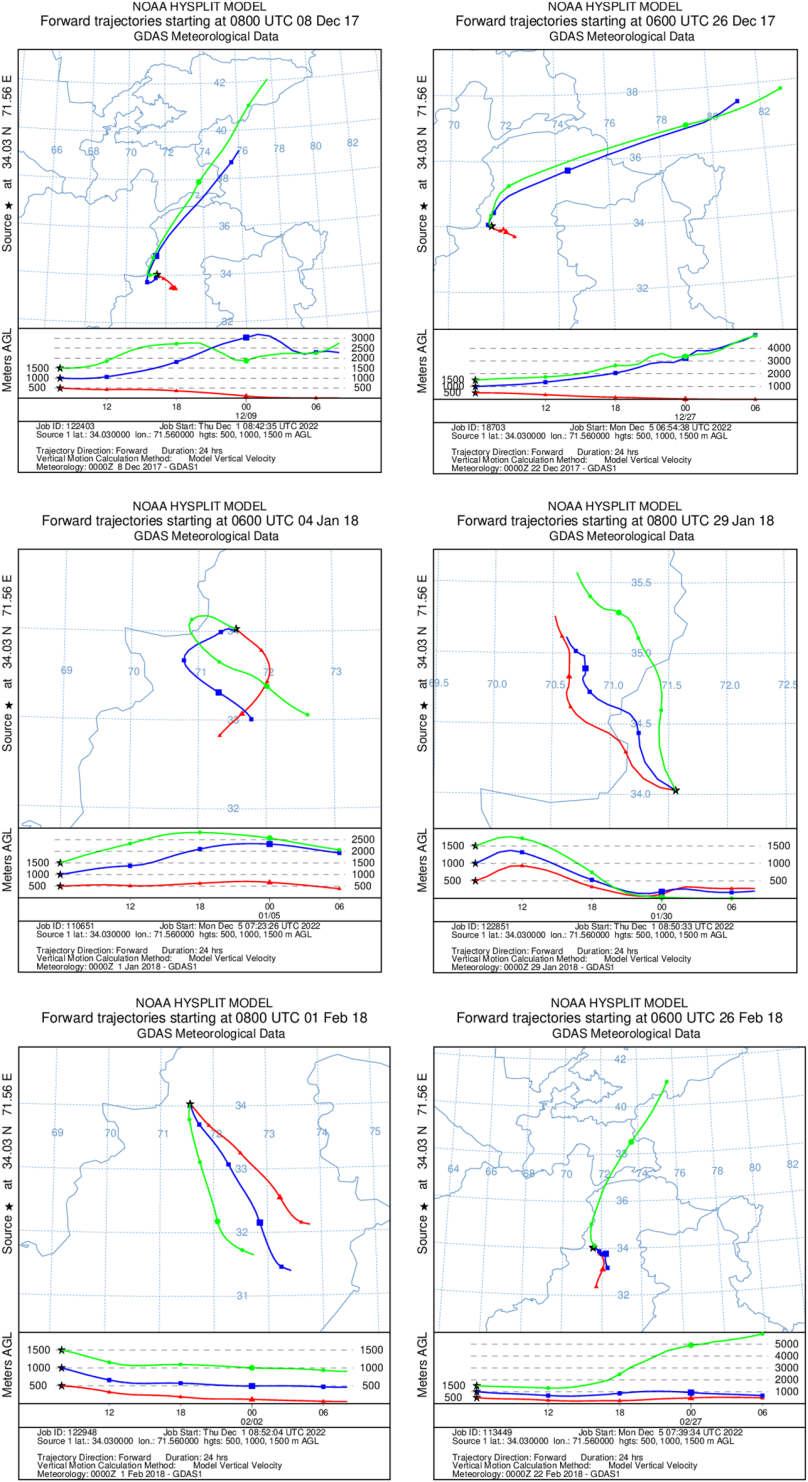
Back trajectories of air masses showing possible long-range transport.

The sources and paths of the chemical constituents in the atmospheric aerosols were determined using air mass backward trajectory analysis. The Hybrid Single-Particle Lagrangian Integrated Trajectory (HYSPLIT) model from the National Oceanic and Atmospheric Administration (NOAA) was used to simulate air mass return trajectories^126^. At noon (local time), three heights—500, 100, and 1500 m were chosen for the air mass trajectories to be simulated (Fig. 7).

## Conclusions

The results of the current research study reveal that PM_10_ concentration (586 µg m^−3^) from December 2017 to February 2018 exceeds the standard limit established by both WHO (45 µg m^−3^) and NEQSAA (150 µg m^−3^) over the study location. The values of PM_10_ mass concentration are found to be higher than the values measured in other locations of Pakistan and other global cities. Prominent water-soluble ions detected were Ca^2+^, Na^+^, K^+^, NH_4_^+^, and Mg^2+^, with Ca^2+^ being most abundant due to its association with dust, which is a prominent contributor to the coarse aerosol fraction. The Positive Matrix Factorization model suggested five sources largely contributing to the measured PM_10_ including industrial emissions (accounting for 6.4%), soil and re-suspended dust (18.3%), household combustion (21.1%), metallurgic industries (26.9%), and vehicular emissions (27.3%). Results suggest that meteorology (temperature, RH, WS, and RF) plays a key role in modulating PM_10_ mass concentration. Overall, temperature and relative humidity had positive associations, whereas WS and RF had a negative association with PM_10_, which means that the former encourages high levels of PM_10_. The HYSPLIT model showed that air mass sources impacting the study site included a blend of those originating from distant upwind countries and localized sources.

This study points to continued efforts needed to mitigate extremely high PM_10_ levels, which is complicated owing to influence from natural sources such as dust that are difficult to control and also vulnerable to long-range transport in the region. Installation of air quality monitoring stations at various locations across the country is highly suggested. In the future, it is very necessary to develop effective control strategies and to update and put into practice air quality standards and legislation. Future measurements are encouraged to consider incorporating water-soluble anions (e.g., sulfate, nitrate) and elemental and organic carbon species as those are abundant contributors to PM loadings in major cities. Furthermore, higher time resolution measurements are suggested, as well as more detailed meteorological data analysis including boundary layer dynamics that can impact seasonal PM concentrations in the surface mixing layer. Data gaps for influential variables are encouraged to be filled by reanalysis datasets and potentially also remote sensing data. This study focused on the winter season but for better context future work is warranted to look at the other times of year to understand the full picture of PM_10_ in this region across an annual cycle.

## Data Availability

All data generated or analyzed during this study are included in this published article.
